# The ‘Automated Versatile Modular Reactor’: construction and use

**DOI:** 10.1155/S1463924687000336

**Published:** 1987

**Authors:** Catherine Porte, Dominique Roussin, Jean-Claude Bondiou, Françoise Hodac, Alain Delacroix

**Affiliations:** 1 Laboratoire de Chimie Industrielle Conservatoire National des Arts et Métiers 292 rue Saint Martin Paris Cedex 03 75141 France; 2 Laboratoire de Chimie Industrielle CNAM and Société SALSI Monthyon 77120 France; 3 Société Chimique Pointet Girard 35 Avenue Jean Jaurès Villeneuve la Garenne 92390 France


Journal of Automatic Chemistry/*Journal of Clinical Laboratory Automation*, Vol. 9, No. 4 (October–December 1987), pp. 166–173

### The 'Automated Versatile Modular Reactor': construction and use

**Catherine Porte[1], Dominique Roussin[2], Jean-Claude Bondiou[1], Françoise Hodac[3] and Alain Delacroix[1]**

[1]*Laboratoire de Chimie Industrielle, Conservatoire National des Arts et Métiers, 292 rue Saint Martin, 75141 Paris Cedex 03, France,*
[2]*Laboratoire de Chimie Industrielle CNAM and Société SALSI, 77120 Monthyon, France,*
[3]*Société Chimique Pointet Girard, 35 Avenue Jean Jaurès, 92390 Villeneuve la Garenne, France*

Automation of organic syntheses is a very recent technique in laboratory research in organic chemistry. Because of the complexity of its implementation it was introduced a long time after the automation of analysis systems, which have the advantage of being relatively simple due to their repetitive nature.

Automation of discontinuous elementary operations specific to organic chemistry on a laboratory scale was developed as early as 1970 by the Roussel Uclaf Company [1]. A modular system was chosen, because of the diversity of possible operations. Each module governs a simple operation: measuring and comparing a temperature, controlling a pump, etc. These elements communicate with one another through the on-off signal. The direction is provided by a logical programmable wired system. This modular system, Logilap (R), solves numerous problems in automation [2–4].

The necessity for optimizing syntheses led to their complete automation. It then became necessary to use a stronger logical sequential system and by 1975 microcomputers were used to perform complete syntheses. Different tests were carried out and the following achievements can be mentioned:

In 1983 M. G. Kemp's team [5] at Imperial Chemical Industries (ICI) described the ıesults of studies begin in the 1970s. This system, based on a microcomputer PDP 11, permits the automation of four one-liter reactors independently using shared-time control. This computer system, called CALAB, makes it possible to control pH and temperatures between $-20\,°C$ and $+180\,°C$ and to introduce four liquid reagents in the reactor.

C. E. Berkoff (Smith Kline and French Laboratories) [6] team's goal was the automatic optimization of chemical reactions using the Simplex method. The glass reactor has a capacity of 100 ml; the temperature is programmed; the liquid reagents are introduced by four metering pumps. An on-line analysis, using a HPLC, is linked to the system, which is controlled by a PDP 11.

M. Legrand team's [7] of the Société Roussel Uclaf, developed an automatic system capable of controlling chemical reactions. The 1 l reactor is made out of glass. Heat is provided by a set of heating resistances attached to the exterior of the reactor. The reactor is cooled by immersion in a refrigerated bath placed on a thermovalet. The reaction is controlled by a PDP 11.

These three teams had a similar aim: fixed parameters in order to optimize syntheses in pharmaceutical chemistry. The articles describe research begun in the 1970s and all employed a PDP 11 microcomputer.

More recently F. L. Fuchs and his team [8] at Purdue University described their use of a robot-system, Zymark, created to automate the preparation of samples for chemical and biochemical analysis. The authors adapted this robotic system to implement a simple chemical synthesis and studied the kinetics of the reaction.

In the Conservatoire National des Arts et Metiers (CNAM) Industrial Chemical Laboratory, efforts at early successes were followed by limitations due to controlling systems: the logical correlator was not adapted to the sequential problems which occur frequently in chemistry. Thus, since 1975, the authors have been testing various control apparatus: microprocessor-cards [9], microcomputers [10] and bottom of the range controllers [11–14] numerous one-stage syntheses have since been carried out automatically [2–4, 9–14].

With this range of apparatus adapted to the control of automation, the authors set out to automate syntheses in several stages. To obtain this objective, the following classical operations in chemical industrial syntheses had to be automated:

(1) Stir in a reaction mixture.

(2) Control the temperature of the reaction mixture.

(3) Distill under atmospheric pressure or reduced pressure.

(4) Introduce liquid reagents, solvents or solutions.

(5) Extract products by washing and decanting.

An efficient system must be versatile if it is to allow automation of new chemical experiments. An apparatus adaptable without modification to any experiment was not feasible because of high cost and the difficulty of anticipating the equipment which will be available in the future.

Thus we used a modular structure which permitted modification or elimination of each element and addition of an unlimited number of new modules. This Automated Versatile Modular Reactor (AVMR) was built with commercially available industrial equipment [15]. Therefore, techniques developed in our laboratory can be used in industrial pilot situations.

To test the AMVR, a synthesis which was well-defined and provided an opportunity to link the elementary operations was chosen. The synthesis required adaptation to the various technological problems which arise due to the corrosive effects of different products and/or reagents.

In this article, the operating process of the synthesis, the equipment used to perform each portion of the operation, the main-system of control, and the stages of the automated experiment are described.

### The chemical synthesis

The synthesis chosen is the fabrication of 1,3,4-triphenyl 5-methyl pyrazole (the precursor of the anti-inflammatory drug isofezolac or 1,3,4-triphenyl 5-methyl pyrazole acetic acid).

This synthesis has three steps: (1) the formation of hydrazone form desoxybenzoine in the solvent toluene; (2) the acetylation of the product in pyridine, which traps the HCl in the form of pyridinium chloride; and (3) the cyclization of the acetylated derivative in N-methyl pyrrolidone (NMP) using potassium hydroxide as a catalyst.

The reactions are shown below:

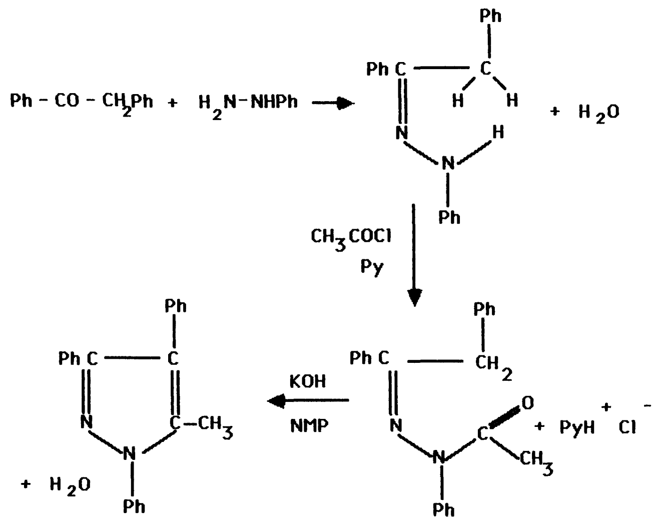

### Pilot operation mode

The synthesis of 1,3,4-triphenyl 5-methyl pyrazole, was done in a 250 l pilot vessel.

*Formation of hydrazone:* the reactor was loaded with 16·5 kg desoxybenzoin, 120 l toluene, and 9·2 kg phenylhydrazin. It was brought to reflux. About 35 l toluene was removed during one to one-and-a-half hours of distillation. Following reflux for an additional hour, formation of hydrazone was confirmed by thin layer chromatography (TLC).

*Acetylation of the hydrazone:* the solution was cooled to 25 °C, and 9·4 kg pyridine were added. 9·3 kg Acetyl chloride was added over a 30 min period during which the temperature was held below 45 °C. The mixture was maintained at 45–50 °C for 4 h and then allowed to cool overnight. On the following day, acetylation of hydrazone was confirmed by TLC. We added 70 l water to wash the organic phase. If the concentrate is not well decanted, it is heated to 50 °C, washed and checked by TLC. The toluene was removed by distillation under reduced pressure.

*Cyclization in a pyrazoled derivative:* 120 l N-methyl pyrrolidone (NMP), 830 g potassium hydroxyde in pastille form, and 830 ml water were added to the concentrate. Following these additions, the temperature of the mixture should rise from 20 °C to 45 °C within 2 h. Formation of the product was monitored by TLC.

The N-methyl pyrrolidone (NMP) was reduced under about 30 mm Hg at a temperature of 105 °C. When the methyl pyrazole begins to crystallize, 1200 ml isopropanol was added. The crystallized produce was stirred overnight. The mixture was then cooled to 0 °C and stirred for an additional hour. The yield or crystalline product was 85%.

### 'GRAFCET' of the experiment

As shown elsewhere [16], the description of chemical operation modes is rationalized by using a 'Grafcet', a description of the schedule of logical and sequential automation steps [17]. The first level of Grafcet is a description of the chemical operations which is transcribed to the chemical operating mode (figure 1). The second level is a technical level detailed description which is translated into the language of the decision system. The time course of the synthesis is given in figure 2.

### Laboratory equipment

*The reactor* (2·5 l) was made of Pyrex glass so that foam and colour changes could be observed. It was jacketed for temperature control and equipped with a valve at the base to permit drainage.

*Temperature regulation* was achieved with a 'Unistat' (Society Huber) thermostatic circulating bath with oil as the heat exchange medium. This system, one of the simplest available, is precise enough for 'classical' experiments in chemistry.

The thermostatic bath as modified for this study had a volume capacity of 8·5 l, a heating capacity of 1500 W, a cooling capacity of 360 W at −10 °C, and a maximum pump flow of 18 l/mn. The temperature threshold is reached and maintained by the Proportional Integral Derivative (PID) regulation specific to 'Unistat'. The temperature threshold can be (1) internal (where only one temperature is possible), or (2) external (where it can be provided by an apparatus with a compatible analogue output [0–10 V for a −200 °C to +800 °C linear scale]).

The threshold varies with time with the PID regulation. The threshold is easily reached for rising temperatures (3·5 °C/mn) but is more difficult when decreasing tem-

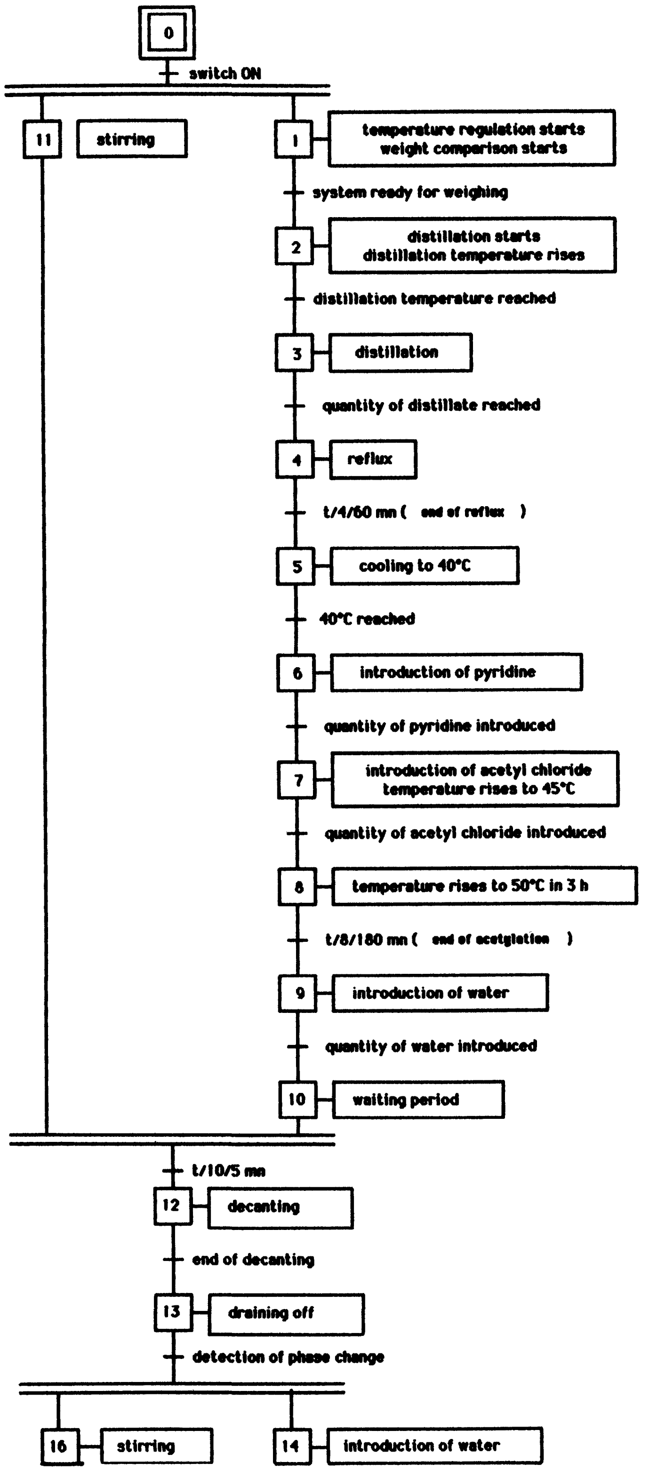

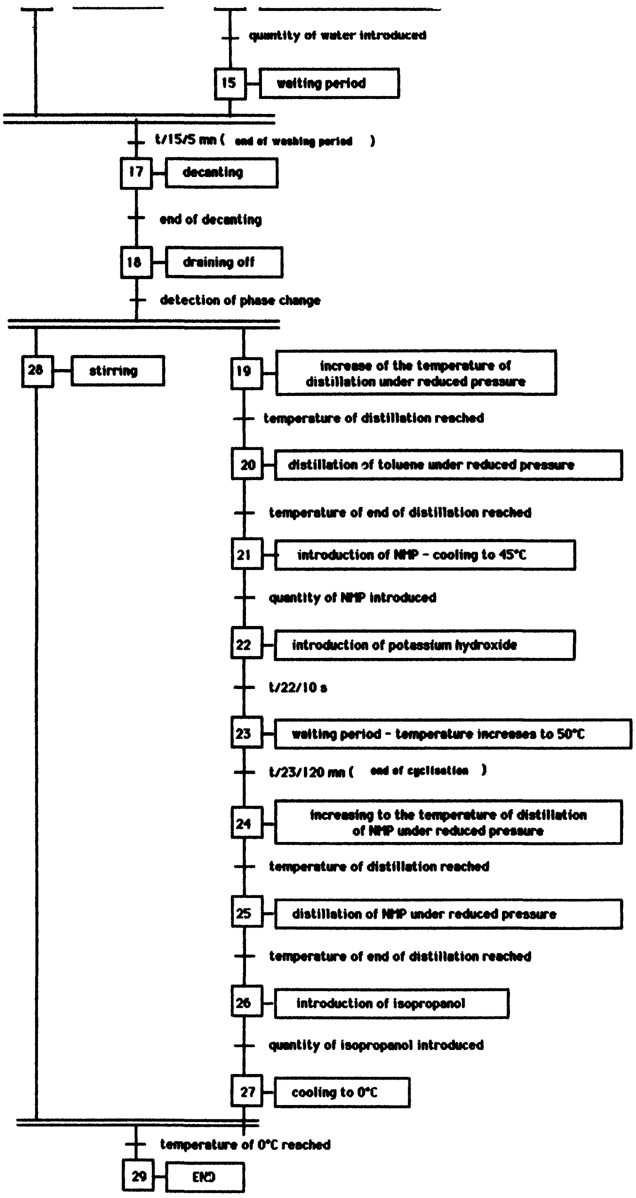

*Figure 1. Grafcet.*

peratures are desired. The use of a compressor, which automatically turns on when the slope is positive and stops when the slope is negative or zero, brings the rate of falling temperature to 1·5 °C/mn.

A programmer is used to provide the analog input signal 'RNZ' (Society Coreci). This apparatus, based on a microprocessor, permits an analog voltage signal (0–10 V) corresponding to a linear temperature range of −200 °C to +800 °C to be produced as well as a four-bit word which sets the logical state of four relays. Furthermore, the programmer has three inputs: $E_1$ (in the on position, interrupts the progress of the logical and analog programs. This enables us to block the programmer in a given position). $E_2$ (this impulse memorizes thresholds and the instantaneous states or relays in the case of mains failure), $E_3$ (an impulse on this input starts the program, which makes automatic remote control possible).

Analog and logical thresholds are programmed separately. 99 segments are used for each channel. The analog part is dominant because of its superior hierarchical level. The final threshold (four-digit word) and the duration (three-digit word) are fixed for each segment.

### *Distillation and reflux device*

The following operations were performed in the pilot operating mode: distillation of a given quantity of azeotrope water-toluene, toluene reflux for one to one-

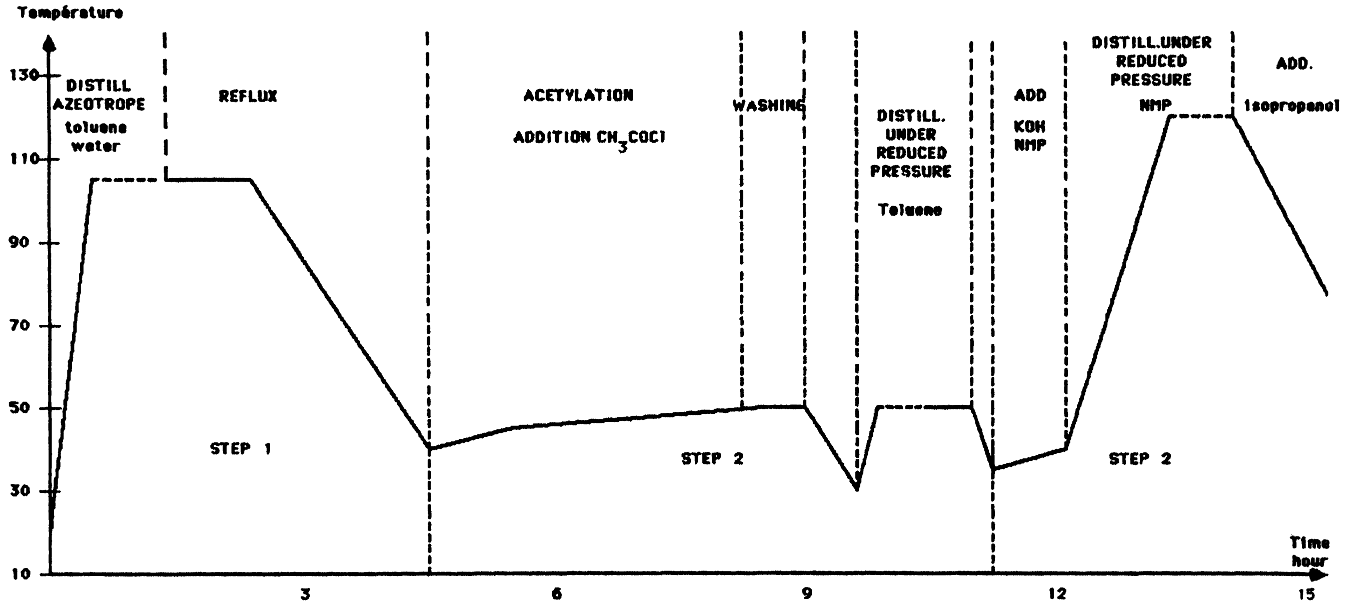

*Figure 2. Time course of the synthesis.*

and-a-half hours, toluene elimination by distillation under reduced pressure, and NMP elimination by distillation under reduced pressure.

These different operations represent various cases of distillation and reflux. The automation of these operations involves the following.

The possibility of passing automatically from distillation to reflux and conversely. Therefore the reactor was equipped with a cooler, which had a still-head at its vase (figure 3). The manual tap was substituted for a magnetic system propelled by a solenoid – this is a common device made up of a glass stick the end of which is rounded and ground and obstructs the passage of the distillate, at the other end there is a magnetic bar. The solenoid maintains the stick in high position during the distillation phases. During the reflux period the glass cane is in low position.

The possibility of distilling under reduced pressure. The reduction in pressure is assured by opening a three-way valve, which is safer than a set of two-way valves as it is impossible, even if there is a breakdown in the control system, for the reactor to become pressurized.

The possibility of weighing the quantity of distillate. One end of the cooler had a two-way valve to convey the distillate towards the balance. Conversely the valve directs the distillate towards a closed container where it is distilled under reduced or atmospheric pressure.

*Device for introducing reagents*

The system must control the introduction of six liquids (reagents or solvents). A system which covers the most applications, and which uses metering pumps, was adopted and this allows reagents to be introduced either according to time, or according to weight.

*According to time:* reliable metering pumps be used. Once the rate of flow is known, a liquid can be introduced in relation to the flow. In this case it is necessary to measure the time taken for a liquid to pass through the pump and to verify that the flow stays the same.

*According to weight:* in addition to the pumps, this requires a balance and a system of comparison in order to know whether or not the threshold is reached. This system is the most expensive but also the most convenient as there is no need to calculate the rate of flow. This is a real advantage in the development stage where the operation modes are not yet fixed.

The advantage of the system according to weight is that it is possible to control the quantity introduced and any error in the weighing operations.

The following were used to introduce the liquids: metering pumps (Society Prominent), electrovalves (Society Fluorocarbon), a balance (Society Mettler) and a multiple threshold comparator (CNAM).

*Metering pumps:* the metering pumps were chosen for their range in the rate of flow and their resistance to corrosion. (Their characteristics are given in figure 4.) Using these pumps the movement of the solenoid can be regulated manually and this makes it possible to change the volume or liquid pumped at each stroke. The frequency of the strokes can also be regulated using a potentiometer in a ratio of 1/25.

The frequency of the impulses is easily controlled by an analog frequency converter (the reliability of this type of operation has already been demonstrated) [13].

Each pump is equipped with a safety valve which makes it possible to obtain a regular flow and to isolate the pump from the reactor under reduced pressure if the force of counter-pressure is sufficient.

*Electrovalves:* Only three pumps were used to introduce the six liquids, electrovalves were placed on each suction circuit (Electovalves DV2 144 – Society Fluorocarbon).

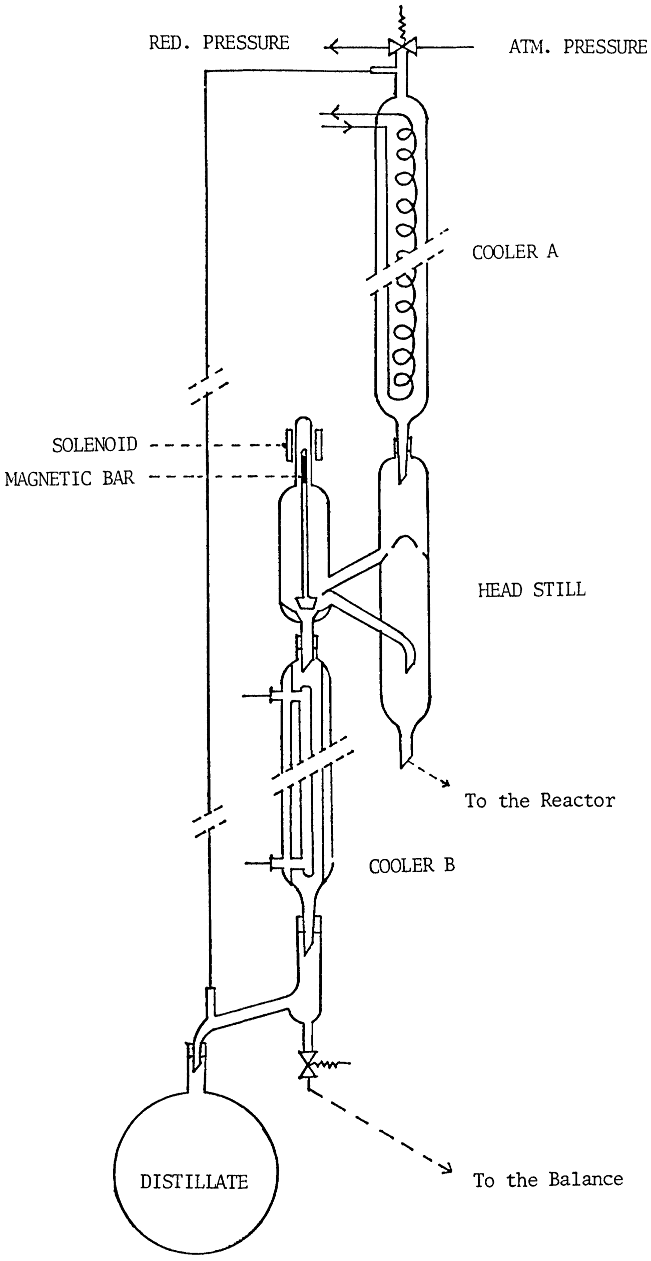

*Figure 3. Distillation and reflux device.*

| | Variable flow | Body | Introduction of: |
|---|---|---|---|
| Pump 1 | 3 to 789 ml/h<br>A 200LS | Stainless steel | Acetyl chloride<br>toluene (washing) |
| Pump 2 | 10 to 1980 ml/h<br>A 1002 T | Teflon | Acetyl chloride<br>Pyridine |
| Pump 3 | 25 to 5760 ml/h<br>B 10006 T | Teflon | Water<br>N-methyl pyrrolidene |

*Figure 4. Characteristics of the pumps.*

*Balance and multiple threshold comparator:* A 16 kg balance (PC 16 – Society Mettler), was used which was equipped with a parallel BCD interface (accurate to $\pm 1/10$ g). The system of comparison used for the balance is the multiple threshold comparator called DRCP, constructed in the authors laboratory and now available for sale [18]. The comparator can control 98 successive automatic weighing operations. It takes into account the weight, the real quantity of liquid to remove (or introduce) form (or on) the balance and after calculating the threshold continually compares it with the real weight.

Its logical input receives an ON/OFF signal from the control system which orders it to calculate the threshold. It has two logical outputs: the first one is used to signal that the threshold is completed, the second one signals the end of comparison.

*Decanting – washing – draining off*

In order to decant, the control system has to direct the stirring rod. In this case, the decanting time is determined beforehand. The two water washings were effected at 50 °C, the temperature at which decantation was most efficient.

The gravity method was chosen for draining off the reactor. In order to automate the draining off, the reator was adapted by adding an electrovalve placed as near as possible to the bottom in order to avoid too much dead volume.

The electrovalve has to be able to withstand aggressive products, ensure that the reactor is waterproof even under reduced pressure, and permit draining off at the right moment. The user is able to choose the material used to construct the most important components of the electrovalve; the body and the diaphragm. A Teflon body is often used in laboratories, despite of its high price, as it is the most adaptable. The authors used a Viton DF 150 diaphragm, as this product is not affected by NMP, pyridine, and acetyl chloride. The weight increase observed with toluene (70%) was not a major drawback as this can be reversed (Electrovalve PE3T 220 – Society S.I.B.).

The system decants well, but in order to drain off suspensions or crystallized products the authors looked for industrial valves. There is nothing presently available for small capacity reactors. (This kind of valve is being constructed in the authors' laboratory.)

For automatic decanting the interface between two phases must be detectable and the authors used a proximity capacity sensor (E2K C25M2 – Society Omron).

*Decision system*

A combinational and sequential programmable controller, the Sysmac P5R 10E (Society Carlo Gavazzi Omron), was chosen. The combination program is a direct translation of the ladder diagram based on Boole algebra. The sequencer is programmed by an automatic device which controls program instructions 'step to step'. The language used is a functional diagram similar to the Grafcet.

*Electric panel*

The combinational output relays can withstand 250 V under 2 A. It is therefore possible to link the automate and actuator directly. Each relay works as a switch on the actuator's supply circuit.

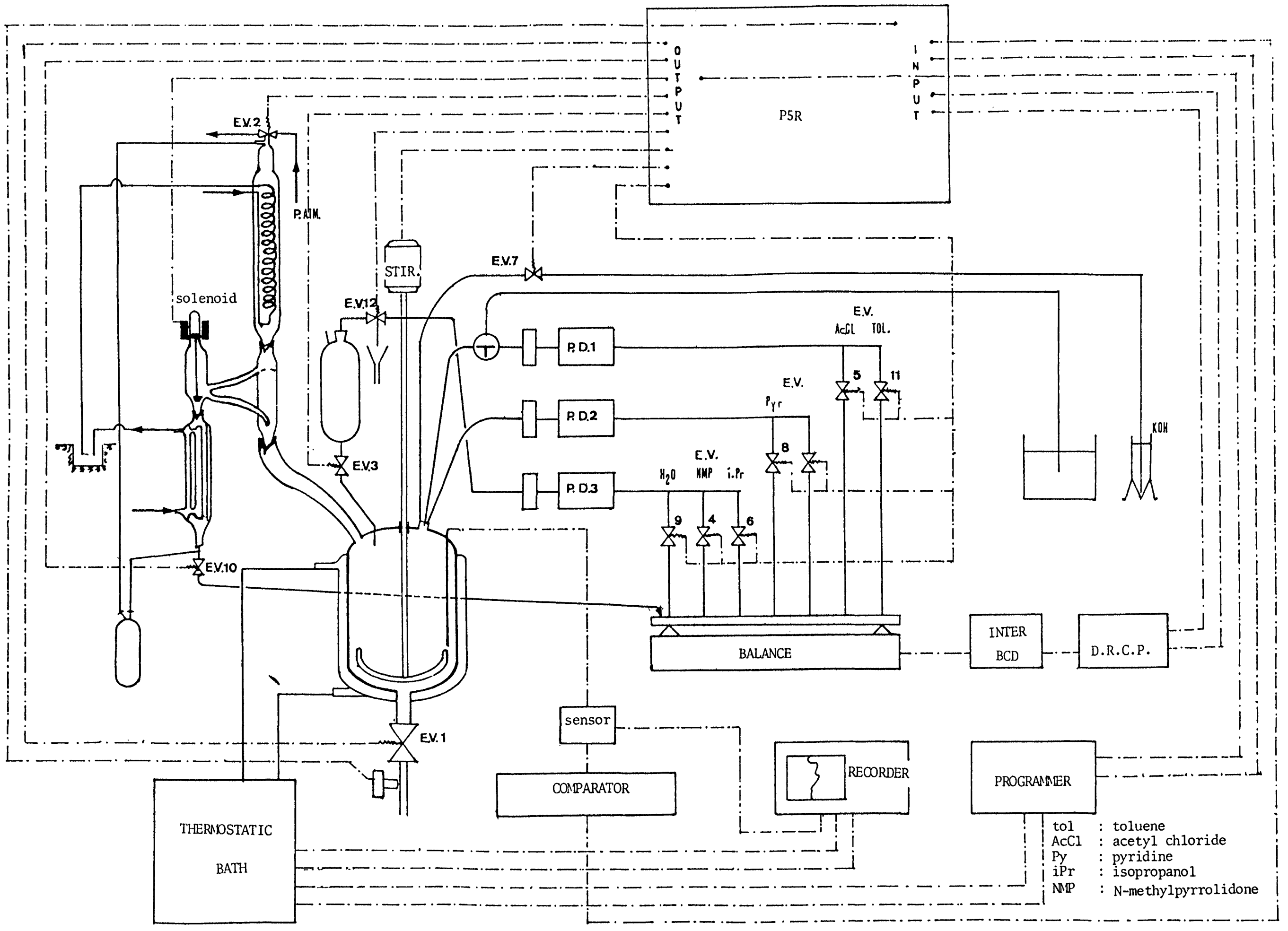

*Figure 5. The Assembled Automated Versatile Modular Reactor.*

A panel capable of supplying 24 actuators with 220 V has been constructed. Each output commands an actuator: either by the combinational, if the corresponding switch is closed, or by a three positions push-button (run, stop, pulse). This enables the program to be constructed or each actuator to be controlled manually. Each actuator has a control light which shows which actuators are in use.

Figures 5 and 6 show the assembled AVMR.

### Automatic run of the experiment

As soon as the start switch is ON, the experiment begins. Then all the operations followed the Grafcet given in figure 1. At the end of automatic experiment, as the electrovalve at the bottom of the reactor is too narrow to let out all the reaction mixture, the reacture is dismantled.

### Results

In the first test of the AVMR 1,3,4-triphenyl 5-methyl pyrazole (the precursor of the anti-inflammatory drug isofezolac or 1,3,4-triphenyl 5-methyl pyrazole acetic acid) was synthesized under conditions thought to be identical to those used in the industrial operating mode. However, the yield was only 68% and not the expected 84%. This discrepancy led us to re-evaluate the system and look for undefined and/or poorly defined operational parameters. In doing so it was noted that the end point of the distillation of the NMP was not described in sufficient detail. At the laboratory bench, experimenters considered that the experiment was over when the mixture turned brown. In order to detect the end of distillation, had the authors initially chosen the medium temperature to be 10 °C above boiling temperature of NMP. It is likely that the remaining residual NMP prevented the product from completely crystallizing. By raising the end point of distillation to 25 °C above boiling temperature a yield of 84% was obtained. Thus with four experiments (figure 7), the operating mode had been standardized and the random bad industrial yields explained.

The usefulness of an automated mode of modular reactor for chemical syntheses has been demonstrated in this article. This modular reactor has the capability of reproducing industrial scale systems, and the results obtained from it could be utilized in industrial scale syntheses.

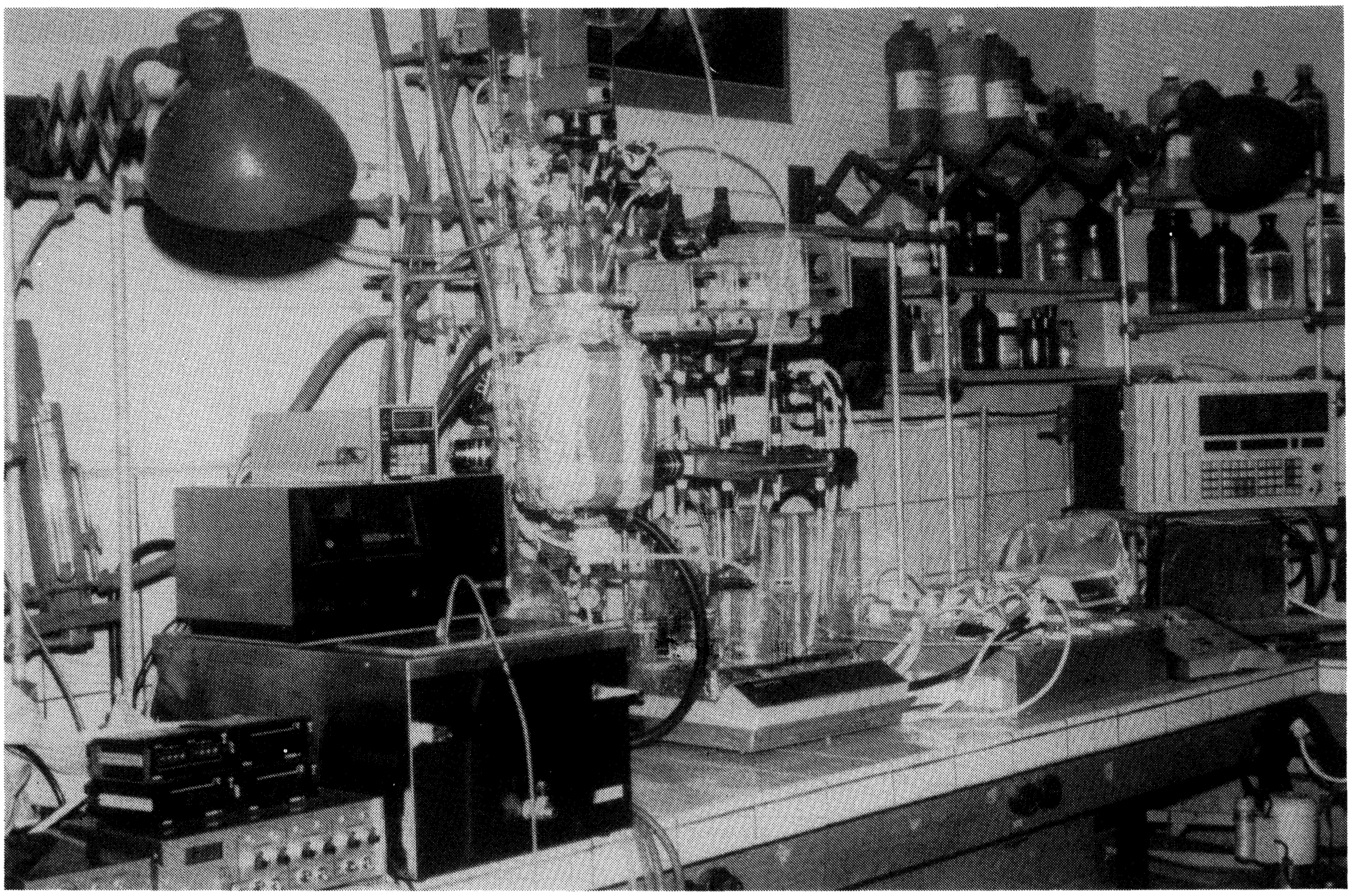

*Figure 6. The Automated Versatile Modular Reactor.*

| Experiment | 1 | 2 | 3 | 4 |
|---|---|---|---|---|
| End distillation Temperature | 125 °C | 125 °C | 140 °C | 145 °C |
| Yield | 68% | 68.5% | 84% | 87% |

*Figure 7. Results of automated experiments.*

### Conclusion

In order to optimize pharmaceutical chemical syntheses, a single stage synthesis had first to be automated. Next a multi-stage synthesis was automated to optimize one-pot syntheses. This is more complicated as the passage from one step to another necessitates different processes (extraction, distillation etc.).

An automatic system was created which was flexible enough to process several succesive and simultaneous elementary operations – the Automated Versatile Modular Reactor.

A relatively complex synthesis in three stages was chosen, it had 29 operations which correspond to about 100 elementary acts, to help construct the reactor.

This synthesis was reproduced in the first automated experiment. This result proves that a number of elementary chemical operations can be linked.

To create the AVMR, the authors looked for, tested and assembled commercial industrial equipment to perfect a specific module.

This prototype is made up of commercial modules linked by standardized interfaces. So it is easy to modify and it can carry out complex chemical syntheses.

We suggest two lines of research for the elaboration of this system:

The first, which is a question of computer science, concerns the programming languages. It seems that the relation between man and machine is a problem for chemists who tend to exaggerate the importance of communications to the detriment of the realization of automatic chemical operations.

This leads us to the second and most important line of research, concerning the automation of more complex chemical synthesis. For this we need new sensors and actuators compatible with the reagents used. This implies a thorough knowledge of the materials in use, a study of the reliability of sensors and of the potential of actuators. This means standardizing information transfer.

### References

1. LEGRAND, M. and FOUCARD, A., *Journal of Chemical Education*, **55** (1978), 767.

2. DELACROIX, A. VELTZ, J.-N. and LE BERRE, A. *Bull. Soc. Chim. Fr.*, **II** (1978), 481.

3. LONCHAMBON, G., DELACROIX, A., PETIT, J. and LECONTE, P., *Bull. Soc. Chim. Fr*, **II** (1981), 71.

4. DEBREUILLE, W., Mémoire Ingénieur CNAM (February 1984).

5. KEMP, M. G., *Chemistry and Industry* (1983), 355.

6. WINCOV, H., SCHAINBAUM, J., BUCKLEGH, J., LONGINO, G., HILL, J. and BERKOFF, C. E., *Analytica Chimica Acta*, **103** (1978), 469.

7. LEGRAND, M. and BOLLA, P., *Journal of Automatic Chemistry*, **7** (1), (1985), 31.

8. FRISBEE, A. R., NANTS, M. H., KRAMER, W. G. and FUCHS, P. L., *Journal of the American Chemical Society*, **106** (1984), 7143.

9. PORTE, C., BORRIN, R., BOUMA, S. and DELACROIX, A., *Bull. Soc. Chim. Fr.*, **I** (1982), 90.

10. PETIT, J., PORTE, C. and DELACROIX, A., *Bull. Soc. Chim. Fr.*, **I** (1983), 63.

11. AIDO, L., PORTE, C. and DELACROIX, A., *Bull. Soc. Chim. Fr.*, **I** (1984), 63.

12. BRUNET, Y., PORTE, C., CRETENOT, C., LEFRANÇOIS, B. and DELACROIX, A., *Informations Chimie*, **24** (1984), 171.

13. KROMYDAS, C., *D.E.A. Chimie Appliquée*, Université Pierre et Marie Curie, Paris VI (June 1984).

14. PORTE, C., DERON, M., SEITE, C., CRETENOT, C., LEFRANÇOIS, B. and DELACROIX, A., *Entropie*, **125** (1986).

15. ROUSSIN, D., Mémoire Ingénieur CNAM, Paris (March 1986).

16. PORTE, C., Thèse Doctorat ès Science Physique, Université Pierre et Marie Curie (June 1985).

17. BLANCHARD, M., in *Comprendre, Maîtriser et Appliquer le GRAFCET*, Cepadues (Toulouse, 1979).

18. PORTE, C., ROUSSIN, D. and DELACROIX, A., *Bull. Soc. Chim. Fr.*, **I** (1985), 34.

### VIDEO – HPLC

*Troubleshooting HPLC Systems* is a video course with three 55-minute tapes covering:

- Principles of troubleshooting.
- Fittings, reservoirs, pumps and injectors
- Columns, detectors and preventive maintenance

Experienced workers will find the course more beneficial than newcomers to HPLC, as a basic understanding of HPLC is assumed.

The complete course costs 3000 Dutch guilders; a demonstration tape is available at 50 Dutch guilders on a prepaid basis only.

*For more information write to Elsevier Science publishers, Video Department, P.O. Box 330, 1000 AH Amsterdam, The Netherlands.* Telex 10704 ESPOM NL.

### NOTES FOR AUTHORS

*Journal of Automatic Chemistry incorporating Journal of Clinical Laboratory Automation* covers all aspects of automation and mechanization in analytical, clinical and industrial environments. The *Journal* publishes original research papers; short communications on innovations, techniques and instrumentation, or current research in progress; reports on recent commercial developments; and meeting reports, book reviews and information on forthcoming events. All research papers are refereed.

### Manuscripts

Two copies of articles should be submitted. All articles should be typed in double spacing with ample margins, on one side of the paper only. The following items should be sent: (1) a title-page including a brief and informative title, avoiding the word 'new' and its synonyms; a full list of authors with their affiliations and full addresses; (2) an abstract of about 250 words; (3) the main text; (4) appendices (if any); (5) references; (6) tables, each table on a separate sheet and accompanied by a caption; (7) illustrations (diagrams, drawings and photographs) numbered in a single sequence from 1 upwards and with the author's name on the back of every illustration; captions to illustrations should be typed on a separate sheet. Papers are accepted for publication on condition that they have been submitted only to this *Journal.*

### References

References should be indicated in the text by numbers following the author's name, i.e. Skeggs [6]. In the reference section they should be arranged thus:

to a journal

MANKS, D. P., *Journal of Automatic Chemistry*, **3** (1981), 119.

to a book

MALMSTADT, H. V., in *Topics in Automatic Chemistry*, Ed. Stockwell, P. B. and Foreman, J. K. (Horwood, Chichester, 1978), p. 68.

### Illustrations

Original copies of diagrams and drawings should be supplied, and should be drawn to be suitable for reduction to the page or column width of the *Journal*, i.e. to 85 mm or 179 mm, with special attention to lettering size. Photographs may be sent as glossy prints or as negatives.

### Proofs and offprints

The principal or corresponding author will be sent proofs for checking and will receive 50 offprints free of charge. Additional offprints may be ordered on a form which accompanies the proofs.

*Manuscripts should be sent to either Dr P. B. Stockwell or Ms M. R. Stewart, see inside front cover.*

